# The Risk of Mycotoxin Contamination and Delay Harvesting on Quality and Technological Parameters of Spring Cereals

**DOI:** 10.1111/1750-3841.71092

**Published:** 2026-04-29

**Authors:** Yuliia Kochiieru, Audronė Mankevičienė, Akvilė Jonavičienė, Lauksmė Merkevičiūtė‐Venslovė, Eimantas Venslovas, Roma Semaškienė, Jūratė Ramanauskienė, Karolina Lavrukaitė, Mykola Kochiieru, Jurgita Cesevičienė

**Affiliations:** ^1^ Institute of Agriculture, Lithuanian Research Centre for Agriculture and Forestry Kėdainiai Lithuania

**Keywords:** falling number, mass per hectoliter, mineral elements, mycotoxins, oats, protein, starch, triticale, viscosity

## Abstract

This study aimed to assess the risk of mycotoxin contamination and delayed harvesting on the quality and technological parameters of spring oats and triticale. Seventy‐two grain samples were collected at three stages from four randomized field replots. Delayed harvesting led to high mycotoxin contamination of spring oats by DON and T‐2 toxin and triticale grains by DON and ZEA and diminished main grain quality indicators, including mass per hectoliter, falling number, and RVA pasting properties. The optimal harvest time, especially in wet weather conditions, is a crucial consideration for viscosity. The correlation analyses between mycotoxins and the quality and technological parameters of oats and triticale grains revealed a trend in which the increasing mycotoxin contamination of spring cereal grains leads to negative consequences on mass per hectoliter, protein, starch, K, Can and Mg in oat grains and a reduction in mass per hectoliter, Mg, viscosity, and falling number values in triticale grains.

**Practical Applications:**

Information on the risk of mycotoxin contamination and harvest delays affecting the quality and technological parameters of spring grain crops will be useful and provide new insights to grain producers, buyers, processors, and other interested professionals on factors that reduce the quality of spring grains.

## 1 Introduction

All over the world, more people are including cereals in their diets (Thielecke et al. 2021). Oats (*Avena sativa* L.) are cereals that are widely used as human food and animal feed and for industrial purposes. In recent years, oats have become more popular among people due to their valuable nutritional properties and therapeutic potential for human health (Paudel et al. 2021; Teixido‐Orries et al. 2025).

Starch is the main component of oat grains; its content directly depends on the variety and growing conditions. Refined oatmeal contains 15%–18% protein, 59%–70% sugars and starches (Capouchová et al. 2021; Németh et al. 2021). Research by Capouchová et al. (2021) and Németh et al. (2021) has also shown a wide range of fat contents among oat cultivars, varying from 4.76 to 8.54 g per 100 g. The pasting properties of oat flour are mainly determined by oat starch (Qian et al. 2020).

Global production of triticale is increasing due to rising demand in the food market. Triticale (× *Triticosecale* Wittmack) is a cereal developed by crossing rye (male) and wheat (female) to improve the production and biochemical composition of parental species (Gagiu 2018). The quality of the kernel is a complex of physicochemical characteristics, the manifestation of which depends on its genetic nature and environmental influences (Johansson 2002).

Triticale is currently a recognized crop worldwide, with interest focused on two potential uses: as grain and as a forage plant. Triticale grains, which are characterized by high starch and low protein content, are also one of the raw materials for bioethanol production (Janusauskaite et al. 2019). The economic importance of triticale is reflected in the global areas of approximately 3.6 million ha and production of 14.2 million tons in 2022 (FAOSTAT 2024).

Cereals play an important role in the consumption of macro‐ and micronutrients, and their active consumption, especially whole grains, is associated with a reduced risk of developing several chronic diseases (Sette et al. 2017; Thielecke and Nugent 2018). The human diet is often deficient in minerals such as phosphorus (P), potassium (K), calcium (Ca), and magnesium (Mg). Therefore, there is a significant need for cereals containing high and bioavailable concentrations of these mineral elements (Biel et al. 2020; Hejcman et al. 2013).

The rapid visco analyzer (RVA) has been widely used and is well known for assessing the pasting properties of flour and starch. In the grain industry, the main application of RVA is to analyze the impact of rain damage on the quality of grain (germination) at the delivery point. In recent years, the use of this equipment has also been extended to other areas, such as modeling commercial processing conditions, assessing the effects of pH and temperature on starch gelatinization, and detecting significant changes in viscosity (Cozzolino 2016).

The starch content and flour yield of triticale are comparable to those of wheat, but triticale has a higher ash content (Watanabe et al. 2019). Triticale grain mass per hectoliter (50–74 kg hL^−1^) is lower than that of wheat (73–78 kg hL^−1^), partly due to the different density and wrinkling of the outer bran surface of many triticale varieties (Okuyama, Paulo, et al. 2020; Okuyama, Riede et al. 2020).

A significant defect of triticale is its tendency to sprout during harvest due to its rye rather than wheat origin. Thus, for example, testing of seedlings for damage by falling number (FN) is a prerequisite for compliance with special use requirements. Low FN results may also be due to late‐maturity α‐amylase (LMA) (it is also known as prematurity α‐amylase [PMAA] in Europe) (McGoverin et al. 2011; Wrigley and Bushuk 2017; Zhu 2018).

An important factor affecting the safety of agricultural crops as raw materials for the food and feed industries is natural toxins, such as mycotoxins (Marc 2022). With approximately 25% of food crops affected by fungi and mycotoxins, according to the FAO, food losses are estimated at approximately 1 billion tonnes per year, resulting in multibillion‐dollar economic losses worldwide each year (Neme and Mohammed 2017; Thielecke and Nugent 2018). The main *Fusarium* mycotoxins that can be detected in cereal grains and cereal‐based products are deoxynivalenol (DON) (found mostly in wheat, corn, barley, oats, and rye), T‐2 and HT‐2 toxins (found mainly in oats, wheat, and barley), and zearalenone (ZEA) (found mainly in corn and wheat) (Girolamo et al. 2020; Kovač et al. 2022; Twarużek et al. 2013; Venslovas et al. 2022). Depending on environmental conditions, storage conditions, and plant variety, the type and quantity of mycotoxins produced by a single fungal species may vary from year to year (Igrejas et al. 2020). In addition to the toxicological risk associated with mycotoxin contamination of cereals, grain quality can be compromised because *Fusarium* rot (FHB) infection can affect grain components such as starch and proteins and therefore affect the quality characteristics of grain end use, as well as pasting properties and baking characteristics (Kreuzberger et al. 2015; Schmidt et al. 2017). Harvesting time was found to influence the FN and wet gluten in winter cereals when the weather conditions of the growing season had been rainy (Cesevičienė and Mašauskienė 2007). In Lithuania, the levels of DON, T‐2 toxin, HT‐2 toxin, and ZEA in spring barley grains did not significantly change with delayed harvesting; however, it was determined that the ZEA content increased with delayed harvesting when the growing season was dry and warm and the harvest period was rainy and cool (Venslovas et al. 2024). In addition, the impact of delayed spring wheat harvesting was significant when rainy and cool weather prevailed during the flowering and harvesting period, leading to high levels of mycotoxin contamination, while in other years, when the ripening period was dry, harvest timing had no significant impact (Kochiieru et al. 2021). In Sweden, scientists conducted studies on the dynamics of contamination of grain crops between 2004 and 2018 and found that delayed harvesting was strongly associated with increased levels of DON and ZEN in several crops, while harvest date did not affect levels of the toxins NIV, HT‐2, and T‐2 (Karlsson et al. 2023).

Previous studies reported the influence of harvesting time and meteorological conditions on the occurrence of *Fusarium* species and mycotoxin contamination of spring cereals (Kochiieru et al. 2020), the impact of harvesting time on *Fusarium* mycotoxins in spring wheat grain and their interaction with grain quality (Kochiieru et al. 2021).

Hence, considering the ongoing discussion within the European Commission about regulatory limits for certain mycotoxins, the aim of this study is to determine the risk of mycotoxin contamination and delay harvesting on the quality and technological parameters of spring oats and triticale: mass per hectoliter, protein, fat, starch, ash, K, Ca, Mg, P, viscosity, and FN.

## 2 Materials and Methods

### 2.1 Samples

Spring oat (*A. sativa* L.) and spring triticale (× *Triticosecale* Wittmack) grain samples were harvested in 2016–2018 at the experimental site of the Institute of Agriculture, Lithuanian Agriculture and Forestry Research Centre. A total of 72 grain samples were taken from four randomized field replots in three stages: when the crop reached full maturity (BBCH 89), 10 ± 2 days, and 17 ± 3 days after the first harvest (Table 1).

**TABLE 1:** The oats and triticale grains harvesting time (2016–2018).

Spring cereals	First harvesting time	Second harvesting time	Third harvesting time
	August 4, 2016	August 16, 2016	August 24, 2016
Oats	Full maturity^a^	Full maturity + 12 days	Full maturity + 20 days
Triticale	Full maturity^a^	Full maturity + 12 days	Full maturity + 20 days
	August 31, 2017	September 8, 2017	September 14, 2017
Oats	Full maturity^a^	Full maturity + 8 days	Full maturity + 14 days
Triticale	Full maturity^a^	Full maturity + 8 days	Full maturity + 14 days
	August 6, 2018	August 16, 2018	August 23, 2018
Oats	Full maturity^a^	Full maturity + 10 days	Full maturity + 17 days
Triticale	Full maturity^a^	Full maturity + 10 days	Full maturity + 17 days

^a^ Full maturity (harvesting time at BBCH 89).

In accordance with the recommendations for cultivation technology, fertilizers were applied, and spraying with herbicides, growth regulators, fungicides, and insecticides was carried out.

Grain moisture content was determined after harvesting and before analysis according to ISO 712 (Table 2).

**TABLE 2:** Grain moisture at harvest and before analysis (2016–2018).

Harvesting time	Grain moisture, %					
	At harvest	Before analysis	At harvest	Before analysis	At harvest	Before analysis
	2016		2017		2018	
Spring oats						
First^a^	12.80	12.75	17.35	11.50	11.08	11.08
Second^b^	12.70	12.70	24.38	12.60	18.10	13.13
Third^c^	12.48	12.30	23.73	13.10	12.85	12.60
Spring triticale						
First^a^	14.03	12.02	18.53	12.90	13.90	12.40
Second^b^	13.70	12.25	24.08	13.00	21.90	13.00
Third^c^	13.58	12.04	26.90	14.00	13.70	13.10

^a^ Full maturity (BBCH 89).

^b^ Full maturity + 10 ± 2 days.

^c^ Full maturity + 17 ± 3 days.

### 2.2 Quantification of Mycotoxins

Quantitative assessment of DON, ZEA, and T‐2 was conducted utilizing a commercial Enzyme Linked Immunoassay (ELISA) kit (RIDASCREEN FAST for DON Art. No. R5901; for ZEA Art. No. R5502; for T‐2 Art. No. R5302). This approach relies on the interaction between antibodies and antigens and has been validated by the AOAC Research Institute (ISO 9001 certified Quality Management System).

Reagents supplied with the ELISA (RIDASCREEN, R‐Biopharm, Germany) test kits (microtiter plate with 48 wells, conjugate, antibody, substrate/chromogen, stop solutions, washing buffer) were used in this study for mycotoxin determination. Methanol (CH_3_OH) was obtained from Merck (Darmstadt, Germany). Standard solutions for DON, T‐2, and ZEA, used in constructing the calibration curve, were prepared at concentrations of DON—0, 222, 666, 2000, 6000 (µg kg^−1^); T‐2—0, 50, 100, 200, 400 (µg kg^−1^); and ZEA—0, 50, 100, 200, 400 (µg kg^−1^), all included in the ELISA test kit.

### 2.3 Preparation of Samples and Test Procedure

Five hundred grams of oats and triticale were milled using a 3100 laboratory mill equipped with a 0.8 mm sieve (Perten Instruments). The resulting crushed samples were thoroughly mixed to homogenize them before weighing. The samples were then separated for mycotoxin analysis. Mycotoxin extraction and testing procedures were performed according to the manufacturer's instructions. Distilled water was used for DON extraction, while a methanol–water mixture (70:30 by volume) was employed for ZEA and T‐2. The assay is based on antigen–antibody reactions, in which the wells of a microtiter plate are coated with antibodies specific for each mycotoxin. The introduction of standard solutions or samples occupied antibody binding sites proportionally to the concentration of mycotoxins. Then, the enzyme conjugate and antibodies to mycotoxins were added. Unbound enzyme conjugate was removed during the wash procedure, and substrate/chromogen was added to produce a blue product that turned yellow upon the addition of stop solution. Optical density readings were obtained using a Multiskan Ascent multichannel photometer (Thermo Electron Corp., Vantaa, Finland) with built‐in software at a 450 nm wavelength. Calibration curves were constructed based on standard concentrations and percentage inhibition. The coefficients of determination (*r* ^2^) for DON, ZEA, and T‐2 ranged from 0.988 to 0.998, 0.982 to 0.998, and 0.980 to 0.996, respectively. Absorbance readings were automatically converted to mycotoxin concentrations (µg kg^−1^). The results were evaluated considering the limit of detection (LOD) as per manufacturer guidelines: DON—100.0 µg kg^−1^ (ppb), ZEA—17.0 µg kg^−1^ (billion), T‐2—20 µg kg^−1^ (billion). The evaluation of food and feed safety data referred to EU document No. 2023/915 for DON and ZEA and global research guidelines for T‐2 (European Commission 2013, 2023).

### 2.4 Quality Analyses

The hectoliter weight (HLW) of grain represented the grain weight within a specific volume (ISO 7971‐2). Nitrogen (N), phosphorus (P), potassium (K), calcium (Ca), and magnesium (Mg) contents were assessed in the sulfuric acid digestates. The total N content was determined using the Kjeldahl method with a Kjeltec 1002 system (Tecator AB, Hoganas, Sweden). For crude protein calculation, N content was multiplied by conventional factors—5.7 for triticale and 6.25 for oats (ISO 20483). Starch analysis was conducted via polarimetry using ADP 410 (Bellingham & Stanley, UK) following the ICC 123/1 method with slight modification. Fat extraction utilized the Soxhlet method (ISO 11085) with petroleum ether as the solvent. Ash content was determined through incineration at 550°C (AOAC 923.03). Phosphorus content was quantified spectrophotometrically through a color reaction with ammonium molybdate–vanadate (ISO 6491) at a wavelength of 430 nm on a Cary 50 UV‒Vis spectrophotometer (Varian Inc., Palo Alto, CA, USA). Potassium, calcium, and magnesium contents were determined via atomic absorption (AAnalyst 200, Perkin Elmer, Waltham, MA, USA) following internationally recognized procedures for mineral analysis (ISO 6869). FN analysis for triticale was conducted using FN 1500 equipment (Perten Instruments, Hägersten, Sweden) through the Hagberg method described in ICC 107/1. The pasting properties of whole meals were assessed using the RVA (Tech Master, Newport Scientific, Warriewood, Australia) controlled with the Thermocline software program. The analysis adhered to a 13‐min standard RVA profile (STD1), utilizing a 160‐rpm rotor speed and programmed heating—cooling cycle (50°C–95°C–50°C) as per ICC 162. Recorded parameters included viscosity peak, time and pasting temperature (at rise in viscosity), trough (minimum viscosity at 95°C), breakdown (difference of peak and trough viscosity), final viscosity (viscosity at 50°C), and setback (difference of final and trough viscosity).

All grain characteristics in each sample were determined in two to three replicates. Analyses were performed at the Chemical Research Laboratory of the Institute of Agriculture, LAMMC.

### 2.5 Weather Conditions

**FIGURE 1:**
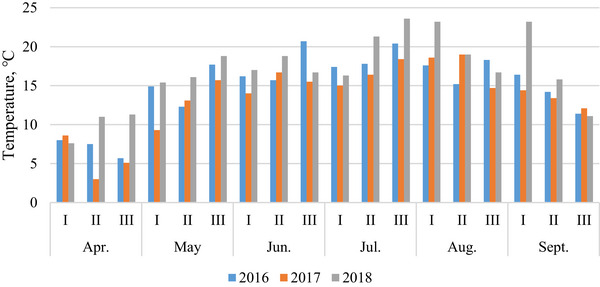
Decadal mean air temperature within each month during the 2016–2018 growing seasons.

**FIGURE 2:**
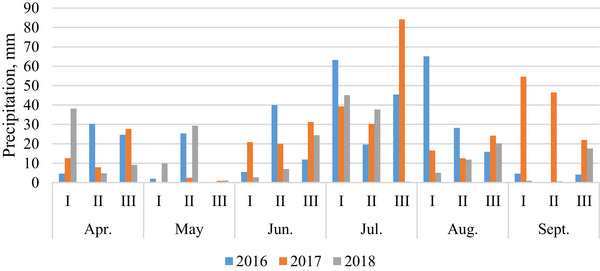
Sum of decadal precipitation within each month during the 2016–2018 growing seasons.

Based on meteorological observations from the local station in Dotnuva (55°23′49.0″ N, 23°51′55.0″ E, Kėdainiai District), the weather patterns in 2016, 2017, and 2018 exhibited distinct characteristics (Figures 1 and 2).

The summer of 2016 was marked by strong winds and heat. June, critical for oat and triticale flowering, was characterized by mostly warm and dry weather. However, the rains that began in August created difficulties during the harvest.

The following year, 2017, presented a completely different picture. June, a critical period for oat and triticale flowering, was marked by cooler weather and prolonged rains. The unfavorable weather persisted until September, and the heavy rainfall complicated the harvest.

In contrast, in 2018, temperatures in the summer months were above normal. The critical flowering phase of oats and triticale in June was characterized by exceptionally warm and dry weather. August brought favorable weather conditions, facilitating a smoother harvest.

### 2.6 Statistical Analysis

The experimental data underwent statistical analysis utilizing SAS, version 9.4 (SAS Institute Inc., USA). Significant differences among the samples were determined using one‐way analysis of variance (ANOVA) followed by Duncan's test. The results with a significance level of p≤0.05 were considered statistically significant. In addition, Pearson correlation analysis was conducted to explore the quantitative relationships between the various grain characteristics.

## 3 Results

### 3.1 Mycotoxin Contamination

In most oat grain samples collected at all harvesting times in 2016 and 2018, mycotoxin levels were low or below the LOD, although higher concentrations were occasionally observed (Table 3).

**TABLE 3:** DON, T‐2, and ZEA occurrence and concentrations in spring oats harvested in 2016–2018.

Harvesting time	No. of analyzed samples	Positive samples, %	Samples exceeding EU regulatory limits, %	Min, µg kg^−1^	Max, µg kg^−1^	Mean, µg kg^−1^	SD, µg kg^−1^
DON							
2016							
First^*^	4	0	0	< LOD	< LOD	< LOD	< LOD
Second^**^	4	0	0	< LOD	< LOD	< LOD	< LOD
Third^***^	4	0	0	< LOD	< LOD	< LOD	< LOD
2017							
First^*^	4	100	0	246.00	784.00	529.00^b^	190.00
Second^**^	4	100	75	490.00	2150.00	1582.00^a^	695.00
Third^***^	4	100	25	538.00	2033.00	970.00^ab^	636.00
2018							
First^*^	4	0	0	< LOD	< LOD	< LOD	< LOD
Second^**^	4	0	0	< LOD	< LOD	< LOD	< LOD
Third^***^	4	0	0	< LOD	< LOD	< LOD	< LOD
T‐2							
2016							
First^*^	4	100	0	60.90	128.20	83.90^a^	27.62
Second^**^	4	100	0	43.60	75.80	59.70^a^	13.80
Third^***^	4	100	0	43.90	104.00	69.10^a^	23.40
2017							
First^*^	4	100	50	102.67	280.00	202.00^a^	81.03
Second^**^	4	100	0	103.00	133.00	124.00^a^	12.88
Third^***^	4	100	50	119.67	268.33	182.00^a^	62.88
2018							
First^*^	4	0	0	< LOD	< LOD	< LOD	< LOD
Second^**^	4	0	0	< LOD	< LOD	< LOD	< LOD
Third^***^	4	0	0	< LOD	< LOD	< LOD	< LOD
ZEA							
2016							
First^*^	4	0	0	< LOD	< LOD	< LOD	< LOD
Second^**^	4	0	0	< LOD	< LOD	< LOD	< LOD
Third^***^	4	100	0	15.50	41.03	24.94	6.91
2017							
First^*^	4	75	0	< LOD	33.00	21.50^a^	6.99
Second^**^	4	100	0	23.00	129.00	54.50^a^	42.63
Third^***^	4	75	0	< LOD	35.00	26.80^a^	8.02
2018							
First^*^	4	25	0	< LOD	24.90	24.90^a^	18.89
Second^**^	4	75	0	< LOD	59.65	28.83^a^	22.15
Third^***^	4	75	0	< LOD	35.40	22.95^a^	9.03

*Note*: Values followed by the same letter in a column within the same year are not significantly different (Duncan's multiple‐range test, *p* < 0.05).

Abbreviations: LOD, limit of detection; SD, standard deviation.

* Full maturity (BBCH 89).

** Full maturity + 10 ± 2 days.

*** Full maturity + 17 ± 3 days.

In the 2016 harvest year, the DON concentrations in 100% of the collected samples were less than the LOD. T‐2 levels were found in 100% of oat samples harvested at all harvest times, and concentrations varied from 43.60 to 128.20 µg kg^−1^. The ZEA concentrations at the first two harvests were below the LOD; however, at the latest harvest, ZEA was detected in all of the samples. The content of ZEA in oat samples varied from 15.50 to 41.03 µg kg^−1^. In 2017, almost 100% of harvested oat grains were contaminated with all tested mycotoxins. The influence of oat harvest time was noticeable only for DON content, which is not the case for T‐2 and ZEA. In 2018, the DON and T‐2 concentrations did not reach the LOD, and the ZEA level varied from < LOD to 59.65 µg kg^−1^.

The triticale grains harvested in 2016 at all harvesting times were 100% contaminated with DON, while T‐2 and ZEA concentrations were below the LOD (Table 4).

**TABLE 4:** DON, T‐2, and ZEA occurrence and concentrations in spring triticale harvested in 2016–2018.

Harvesting time	No. of analyzed samples	Positive samples, %	Samples exceeding EU regulatory limits, %	Min, µg kg^−1^	Max, µg kg^−1^	Mean, µg kg^−1^	SD, µg kg^−1^
DON							
2016							
First^*^	4	100	0	380.00	747.00	520.00^a^	114.60
Second^**^	4	100	0	496.00	953.00	672.00^a^	140.27
Third^***^	4	100	0	485.00	825.00	607.00^a^	127.36
2017							
First^*^	4	100	100	3850.00	6106.00	4583.00^b^	851.12
Second^**^	4	100	100	10,917.00	16,611.00	13,579.00^ab^	1854.62
Third^***^	4	100	100	13,752.00	18,624.00	16,005.00^a^	1878.62
2018							
First^*^	4	0	0	< LOD	< LOD	< LOD	< LOD
Second^**^	4	0	0	< LOD	< LOD	< LOD	< LOD
Third^***^	4	0	0	< LOD	< LOD	< LOD	< LOD
T‐2							
2016							
First^*^	4	0	0	< LOD	< LOD	< LOD	< LOD
Second^**^	4	0	0	< LOD	< LOD	< LOD	< LOD
Third^***^	4	0	0	< LOD	< LOD	< LOD	< LOD
2017							
First^*^	4	75	0	< LOD	91.00	61.00^a^	28.89
Second^**^	4	100	0	53.00	97.00	81.00^a^	16.23
Third^***^	4	100	0	81.00	95.00	89.00^a^	4.93
2018							
First^*^	4	0	0	< LOD	< LOD	< LOD	< LOD
Second^**^	4	0	0	< LOD	< LOD	< LOD	< LOD
Third^***^	4	0	0	< LOD	< LOD	< LOD	< LOD
ZEA							
2016							
First^*^	4	0	0	< LOD	< LOD	< LOD	< LOD
Second^**^	4	0	0	< LOD	< LOD	< LOD	< LOD
Third^***^	4	0	0	< LOD	< LOD	< LOD	< LOD
2017							
First^*^	4	100	0	26.00	106.00	55.00^b^	31.64
Second^**^	4	100	100	419.00	881.00	644.00^a^	132.28
Third^***^	4	100	100	455.00	1079.00	793.00^a^	221.87
2018							
First^*^	4	75	0	< LOD	69.44	41.90^a^	14.44
Second^**^	4	50	0	< LOD	47.18	22.70^a^	16.12
Third^***^	4	75	0	< LOD	65.03	39.50^a^	16.43

*Note*: Values followed by the same letter in a column within the same year are not significantly different (Duncan's multiple‐range test, *p* < 0.05).

Abbreviations: LOD, limit of detection; SD, standard deviation.

* Full maturity (BBCH 89).

** Full maturity + 10 ± 2 days.

*** Full maturity + 17 ± 3 days.

The concentrations of DON ranged from 380.00 to 953.00 µg kg^−1^. Due to delays in harvesting, DON levels in some grain samples were close to the EU maximum permitted level of 1000 µg kg^−1^. In 2017, harvested triticale grains at all harvesting times were contaminated by DON, T‐2, and ZEA. The DON content in all triticale grains collected at all stages of harvesting exceeded the permissible limits. The content of DON in triticale grains ranged from 3850.00 to 18,624.00 µg kg^−1^ and exceeded the permitted level by more than four times. The levels of T‐2 varied from 18.00 to 97.00 µg kg^−1^, while the levels of ZEA varied from 26.00 to 1079.00 µg kg^−1^ across all harvesting times. The concentrations of ZEA exceeded the permitted limit by 8 and 10 times in grains harvested at the second and third harvesting stages, respectively. The impact of delayed harvesting was observed only for DON and ZEA contamination in triticale grains. In 2018, the concentrations of DON and T‐2 in triticale grains harvested at all harvesting times were below the LOD. Only negligible levels of ZEA were detected, and the values did not differ significantly (*p* < 0.05).

### 3.2 Quality Parameters

In 2016, the value of mass per hectoliter in spring oat grains at the first harvesting time was 57.45 kg hL^−1^ and did not differ significantly (*p* < 0.05) across all three harvesting times (Table 5).

**TABLE 5:** The grain mass per hectoliter and contents of protein, fat, starch, ash and its elements in spring oats in 2016–2018.

Harvesting time	Mass per hectoliter, kg hL^−1^	Protein, %	Fat, %	Starch, %	Ash, %	K, %	Ca, %	Mg, %	P, %
	2016								
First^*^	57.45^a^	16.13^a^	7.82^a^	55.88^a^	2.89^a^	0.43^a^	0.18^b^	0.20^a^	0.53^a^
Second^**^	58.78^a^	15.78^a^	8.49^a^	57.06^a^	3.22^a^	0.43^a^	0.20^a^	0.21^a^	0.54^a^
Third^***^	58.18^a^	15.80^a^	8.35^a^	58.04^a^	3.33^a^	0.43^a^	0.18^b^	0.21^a^	0.52^a^
	2017								
First^*^	51.58^a^	16.33^a^	8.46^a^	47.44^a^	2.58^b^	0.46^a^	0.13^b^	0.18^a^	0.54^a^
Second^**^	46.86^b^	14.70^b^	8.44^a^	48.83^a^	3.75^a^	0.43^a^	0.14^a^	0.18^a^	0.54^a^
Third^***^	45.80^b^	15.08^b^	8.26^a^	48.05^a^	3.42^a^	0.44^a^	0.13^b^	0.18^a^	0.54^a^
	2018								
First^*^	58.03^a^	17.00^a^	7.74^b^	54.39^a^	2.31^a^	0.54^a^	0.30^a^	0.26^a^	0.55^a^
Second^**^	55.75^b^	15.95^b^	8.02^ab^	54.11^a^	2.48^a^	0.52^b^	0.27^a^	0.25^a^	0.56^a^
Third^***^	56.70^b^	16.93^a^	8.27^a^	53.39^a^	2.36^a^	0.51^b^	0.28^a^	0.25^a^	0.55^a^

*Note*: Values followed by the same letter in a column are not significantly different (Duncan's multiple‐range test, *p* < 0.05).

* Full maturity (BBCH 89).

** Full maturity + 10 ± 2 days.

*** Full maturity + 17 ± 3 days.

In 2017, the mass per hectoliter at all harvesting times was less than 52 kg hL^−1^, and according to LST 1610:2016, this grain belongs to the second class. In 2018, the flowering period for oats and triticale was characterized by exceptionally warm and dry weather, and the harvest period was warm and dry. In 2018, the mass per hectoliter was 58.03 kg hL^−1^ in oat grains harvested at full maturity, and after the second and third harvesting times, it decreased by 3.92% and 2.29%, respectively. The protein contents decreased by 2.16% and 2.04% in 2016, 9.98% and 7.65% in 2017, and 6.17% and 0.41% in 2018 compared to the initial level at the first harvesting time. It was found that protein content values differed significantly (*p* < 0.05) in oat grains harvested at full maturity from those in grains harvested later in 2017–2018. Due to the delay in harvesting, the starch content increased slightly by 2.07% and 3.72% in 2016 and by 2.85% and 1.27% in 2017, and did not significantly decrease by 0.52% and 1.87% in 2018 compared to the initial levels in oat grains harvested at full maturity. The ash levels increased by 11.42% and 15.22% in 2016, 45.35% and 32.56% in 2017, and 7.35% and 2.16% in 2018 compared to the initial level in grains harvested at full maturity. The fat content increased by 8.57% and 6.77% in 2016 and 3.62% and 6.84% in 2018 compared to the initial level at the first harvesting time. In 2017, a slight decrease in fat levels was observed in oat grains harvested later. The K content in oat grains harvested at all harvesting times in 2016 was 0.43%; in 2017, it varied from 0.43% to 0.46%, and in 2018, it varied from 0.51% to 0.54%. In 2016–2018, the values of Mg ranged from 0.2% to 0.21% in 2016, 0.18% in 2017, and 0.25% to 0.26% in 2018 in grains harvested at all harvesting times, and the values were not significantly different (*p* < 0.05). In oat grains after the second harvest, the Ca content increased by 11% in 2016 and 7% in 2017 compared to its content in grains harvested at full maturity. In 2018, another trend was observed: Ca content decreased in grains harvested at the second and third collections. In 2016–2018, the values of *p* were not significantly different (*p* < 0.05) in oat grains harvested at all harvesting times. However, the content of mass per hectoliter, protein, starch, Ca, and Mg in oat grains was the smallest in 2017 compared to 2016 and 2018.

**FIGURE 3:**
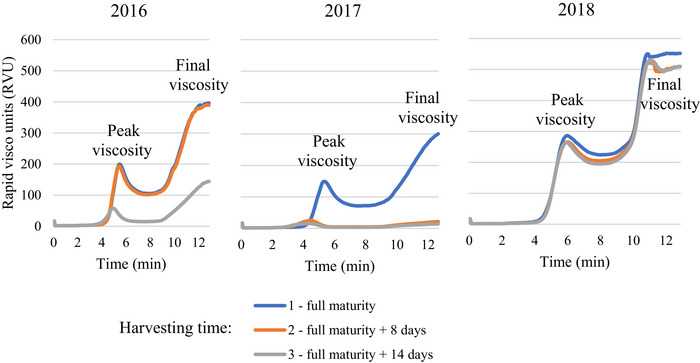
RVA pasting curves for spring oat whole‐meal flour from the grains harvested at different times in 2016–2018.

In two of the three years (Figure 3), the peak, trough, and final viscosities of whole‐meal flour from oat grains harvested at full maturity were essentially higher than those of grains harvested later (*p* < 0.05).

Strong negative correlations were found between DON, T‐2 and oat grain mass per hectoliter (*r* = −0.84; *r* = −0.66) (Table 6).

**TABLE 6:** The correlation between mycotoxins and spring oat grain quality parameters in 2016–2018.

Mycotoxin	Mass per hectoliter	Protein	Fat	Starch	Ash	Viscosity	K	Ca	Mg	P
DON	−0.84^*^	−0.57^*^	0.24	−0.61^*^	0.49	−0.31	−0.30	−0.60^*^	−0.60^*^	−0.07
T‐2	−0.66^*^	−0.43	0.11	−0.62^*^	0.42	0.16	−0.45	−0.79^*^	−0.80^*^	−0.24
ZEA	−0.42	−0.32	0.16	−0.36	0.11	−0.33	−0.12	−0.23	−0.20	0.17

* Significant at *p* < 0.05.

In addition, negative correlations were determined between DON, T‐2, and protein (*r* = −0.57; *r* = −0.43), DON, T‐2, and starch (*r* = −0.61; r = −0.62), DON, T‐2, and K (*r* = −0.30; *r* = −0.45), DON, T‐2, and Ca (*r* = −0.60; *r* = −0.79), and DON, T‐2, and Mg (*r* = −0.60; *r* = −0.8) contents. Weak negative correlations were found between DON, T‐2, and P (*r* = −0.07; *r* = −0.24) and DON, ZEA, and viscosity (*r* = −0.31; *r* = −0.33). Mycotoxin contamination has a strong negative effect on the mass per hectoliter, protein, starch, K, Ca, and Mg in oat grains. The increase in mycotoxin contamination indicates a reduction in grain mass per hectoliter, protein, starch, K, Ca, and Mg parameters.

The nutritional composition of spring triticale grain is outlined in Table 7.

**TABLE 7:** The mass per hectoliter and contents of protein, fat, starch, ash and its elements in spring triticale in 2016–2018.

Harvesting time	Mass per hectoliter, kg hL^−1^	Protein, %	Fat, %	Starch, %	Ash, %	K, %	Ca, %	Mg, %	P, %
	2016								
First^*^	70.55^b^	15.23^a^	2.21^b^	67.94^b^	2.61^a^	0.54^a^	0.17^a^	0.19^a^	0.43^a^
Second^**^	72.35^a^	14.73^b^	2.31^ab^	67.41^b^	2.17^b^	0.53^a^	0.10^b^	0.16^b^	0.41^a^
Third^***^	67.20^c^	14.60^b^	2.50^a^	69.78^a^	2.17^b^	0.52^a^	0.08^b^	0.15^b^	0.42^a^
	2017								
First^*^	69.28^a^	15.90^a^	2.26^a^	66.66^a^	2.00^b^	0.53^a^	0.08^a^	0.17^a^	0.46^a^
Second^**^	65.10^b^	15.78^a^	2.26^a^	67.00^a^	2.25^b^	0.51^a^	0.08^a^	0.16^a^	0.43^b^
Third^***^	60.33^c^	15.88^a^	2.29^a^	67.34^a^	2.67^a^	0.52^a^	0.08^a^	0.16^a^	0.44^ab^
	2018								
First^*^	74.45^a^	15.68^a^	1.79^a^	67.24^a^	2.20^a^	0.53^a^	0.07^a^	0.22^a^	0.43^b^
Second^**^	64.50^c^	15.68^a^	1.66^a^	67.58^a^	2.29^a^	0.52^a^	0.06^a^	0.21^ab^	0.45^a^
Third^***^	70.70^b^	15.73^a^	1.75^a^	66.65^a^	1.92^b^	0.52^a^	0.06^a^	0.20^b^	0.45^a^

*Note*: Values followed by the same letter in a column are not significantly different (Duncan's multiple‐range test, *p* < 0.05).

* Full maturity (BBCH 89).

** Full maturity + 10 ± 2 days.

*** Full maturity + 17 ± 3 days.

Triticale grains harvested later tended to have lower quality compared to those harvested at full maturity. Delayed harvest negatively impacted the grain hectoliter mass value. The mass per hectoliter values significantly (*p* < 0.05) decreased on average by 4.75% in triticale grains harvested at the third harvesting stage in 2016, by 6.03% and 12.92% in 2017, and by 13.36% and 5.03% in 2018 in triticale grains harvested at the second and third stages, respectively. The protein content in 2016 significantly (*p* < 0.05) decreased by 3.28% and 4.14% in triticale grains harvested later, while in 2017 and 2018, the values in grains harvested at all harvest stages were stable. In 2016–2017, there was a trend toward an increase in the fat and starch contents in the triticale grains of the last two harvests, while in 2018, there was a decrease in these indicators in the grain harvested later. The ash content decreased in late‐harvested grains in 2016 and 2018, while in 2017, the values in grains harvested at the second and third harvesting stages significantly (*p* < 0.05) increased. In 2016–2018, the contents of K and P were stable in all harvested triticale grains. However, the levels of Ca and Mg significantly (*p* < 0.05) decreased in the grains harvested at the second and third harvesting stages in 2016, while in the other two years, the values were not significantly different (*p* < 0.05). These triticale trends were consistent with observations in oats.

**FIGURE 4:**
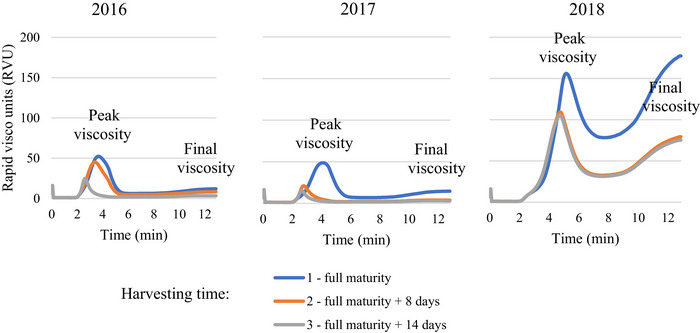
RVA pasting curves for spring triticale whole‐meal flour from the grains harvested at different times in 2016–2018.

**FIGURE 5:**
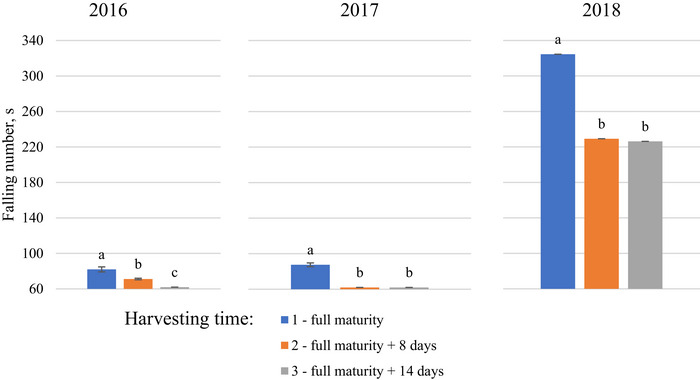
Spring triticale falling number values for the grains harvested at different times in 2016–2018.

Delays in harvest also negatively affected the FN and measured viscosities (Figures 4 and 5). The peak pasting time was consistently highest in spring triticale grains harvested at full maturity across all three research years.

In addition, the peak, trough, and final viscosities of whole‐meal flour from triticale grains harvested at full maturity consistently exceeded those of grains harvested later (*p* < 0.05), especially in the second two years. The minimum value for determining the FN was 62 s, and in two of the three years during the study, some samples reached these values while testing later‐harvested spring triticale grains. Notably, the FN was significantly lower in 2016 and 2017 than in 2018 (*p* < 0.05). However, a consistent pattern emerged each year, with FNs consistently lower in triticale grains harvested later (*p* < 0.05).

In triticale grains, strong negative correlations were found between DON, T‐2, ZEA, and mass per hectoliter (*r* = −0.72; *r* = −0.61; *r* = −0.72, respectively) (Table 8).

**TABLE 8:** The correlation between mycotoxins and spring triticale grain quality indicators in 2016–2018.

Mycotoxin	Mass per hectoliter	Protein	Fat	Starch	Ash	K	Ca	Mg	P	Viscosity	Falling number
DON	−0.72^*^	0.43	0.36	−0.22	0.31	−0.28	−0.12	−0.47	0.07	−0.44	−0.48
T‐2	−0.61^*^	0.52^*^	0.37	−0.31	0.22	−0.07	−0.14	−0.44	0.2	−0.20	−0.48
ZEA	−0.72^*^	0.42	0.24	−0.20	0.32	−0.34	−0.15	−0.37	0.02	−0.50^*^	−0.37

* Significant at *p* < 0.05.

In addition, negative correlations were determined between DON, T‐2, ZEA, and starch (*r* = −0.22; *r* = −0.31; *r* = −0.20), DON, T‐2, ZEA, and K (*r* = −0.28; *r* = −0.07; *r* = −0.34), DON, T‐2, ZEA, and Ca (*r* = −0.12; *r* = −0.14; *r* = −0.15), and DON, T‐2, ZEA, and Mg (*r* = −0.47; *r* = −0.44; *r* = −0.37) contents. Weak negative correlations were found between DON, T‐2, ZEA, and viscosity (*r* = −0.44; *r* = −0.20; *r* = −0.49) and between DON, T‐2, ZEA, and FN (*r* = −0.48; *r* = −0.48; *r* = −0.37) values. Mycotoxin contamination has a strong negative effect on mass per hectoliter, Mg, viscosity, and FN in triticale grains. The increase in mycotoxin contamination suggests a reduction in mass per hectoliter, Mg, viscosity, and FN parameters in triticale grains.

## 4 Discussion

The current investigation was carried out to detect the risk of mycotoxin contamination and delay harvesting on the quality and technological parameters of spring oats and triticale. It was determined that grain quality was highly dependent on environmental growing conditions, particularly meteorological conditions during harvest time, which could cause delays in harvesting and increase the risk of mycotoxin contamination (Kochiieru et al. 2020; El Chami et al. 2023). In the current study, grains of oats and triticale harvested later exhibited higher contamination levels compared to those harvested at full maturity. This trend was particularly pronounced in 2017, characterized by rainy and cool meteorological conditions during vegetation and harvesting periods (Figure 1), where concentrations of mycotoxins (DON and ZEA) in triticale grains significantly surpassed permitted levels, and the level of DON significantly increased in late‐harvested oat grains (European Commission 2023; Kochiieru et al. 2020). However, in 2016, when the flowering period was warm and dry and the harvest period was rainy, only the DON level in late‐harvest triticale grain approached the permissible limit, while mycotoxin contamination of oats was unaffected by harvest time. Similarly, in 2018, a delayed harvest had no significant impact on mycotoxin contamination of oats and triticale when the flowering and harvest periods were warm and dry. In all three years, harvesting time did not impact the T‐2 contamination levels of oats and triticale grains. Karlsson et al. (2023) conducted a study on the dynamics of contamination of grain crops between 2004 and 2018 and found that harvest date did not affect the levels of NIV, HT‐2, and T‐2 toxins. Our investigation showed that oat grains were contaminated more with T‐2 and DON, while triticale was contaminated more with DON and ZEA. In addition, all three mycotoxins (DON, T‐2, and ZEA) were detected in oats and triticale grains when rainy and cool weather conditions were dominant during the vegetation and harvesting periods. Venslovas et al. (2024) determined that the levels of DON, T‐2 toxin, HT‐2 toxin, and ZEA in spring barley grains did not significantly change with delayed harvesting; however, in the same study, it was determined that the ZEA content increased with delayed harvesting when the growing season was dry and warm and the harvest period was rainy and cool. Scientists conducting research in Romania noted that precipitation during the wheat harvest raises the possibility of infection with DON, which is found in the food chain: grain—whole meal flour–bread (Gagiu et al. 2025). They also note that the most significant factor in the contamination of grain crops with fungi and mycotoxins is weather conditions, especially extreme events such as heavy rains, floods, heat waves, and droughts, which are intensifying due to climate change.

It was detected that delays in harvesting oats and triticale resulted in increased ash levels, while grain mass per hectoliter significantly decreased. These observations were particularly pronounced in 2017, when weather conditions were rainy and cool, and the mass per hectoliter in oat grains at all harvesting times was less than 52 kg hL^−1^. According to LST 1610:2016, this grain belonged to the second class. Such grains do not meet quality standards for human consumption and are primarily used as animal feed but may have secondary industrial or low‐grade food uses (Marshall et al. 2013). More ash is also associated with finer grains, the flour of which relatively has more husks, which is also confirmed by the lower mass per hectoliter. In addition, it was determined that the protein content in oats and triticale grains harvested late decreased, while the starch and fat levels were stable. Our results are in line with those of May et al. (2005), who indicated that the timing of harvesting is critical to avoid damage to oat hulls because of the reduction in quality and yield.

In 2016–2018, with delayed oat harvesting, the levels of Mg, P, and Ca remained stable, while the level of K showed a decreasing trend. In triticale grains, with a delay in harvesting, the K, Mg, P, and Ca contents showed a downward trend in all 3‐year studies. Although the impact of harvesting on the quality and technological parameters of oats and triticale has not been sufficiently studied, studies conducted in Lithuania have shown that delayed harvesting of spring barley resulted in an increase in the content of dry matter, crude fat and crude ash and a decrease in the content of crude protein, zinc, and iron, while the levels of Mg, Ca, and P remained relatively stable (Venslovas et al. 2024).

The quality parameters of oats, such as grain mass per hectoliter, protein, starch, Ca, and Mg contents, were the lowest when contamination of grains by mycotoxins (DON, ZEA, and T‐2) was the highest. In addition, El Chami et al. (2023) mentioned that growth in *Fusarium* infection had a negative effect on the quality parameters of wheat, such as protein and gluten content. Contrary to our results, Antes et al. (2001) and Prange et al. (2005) found that strong *Fusarium* infection did not have a significant effect on wheat quality indicators.

Triticale grains showed the lowest mass per hectoliter in 2017 compared to both 2016 and 2018. In oats and triticale, the peak, trough, and final viscosities of whole‐meal flour from grains harvested at full maturity were always higher than those of grains harvested later (*p* < 0.05). In addition, the lowest parameters, the peak, trough, and final viscosities in oats and triticale, were detected in 2017 when cool and rainy weather conditions prevailed during the flowering and harvesting periods. The strong negative correlation between viscosity and mycotoxins showed the negative impact of mycotoxins on RVA pasting properties for spring oats and triticale. In 2017, the moisture of oats and triticale grains at harvest exceeded 17% at the first harvesting stage and 23% and 24% at the second and third harvesting stages, respectively, affecting the quality indicators of the grain. Gagiu et al. (2025) indicated that moisture is important for the microbiological and mycotoxicological safety of wheat grain, flour, and bread, as it affects the physicochemical and rheological properties, as well as the baking process. Deligeorgakis et al. (2023) documented that moisture levels above 14.5% in wheat grain, 14% in flour, and 40% in bread resulted in increased levels of toxigenic fungi of the genera *Fusarium*, *Aspergillus*, and *Penicillium*. The FN influences the dough characteristics and bread properties and indicates preharvest germination.

Delayed harvesting impacted the FN indicator values in all three years of the study. In 2016 and 2017, the FN parameters were the lowest compared to 2018, as rainy weather conditions prevailed during the harvest period in those years. Cesevičienė and Mašauskienė (2007) also documented that FN values in wheat grains harvested later in the rainy period were close to the critical level of 220 s. Tohver et al. (2005) noticed that low FN triticale flour produces a soft dough that can be too runny for bread baking. However, Tohver et al. (2005) detected that weather conditions have a marked effect on this grain quality characteristic. It has been suggested that under wet climate conditions, triticale lines with high FN indicators should be selected (Woś and Brzeziński 2015). The negative impact of mycotoxins on triticale grains in 2016 and 2017 was proven by the negative correlations between mycotoxins (DON, T‐2, and ZEA) and FN indicators. El Chami et al. (2023) also found that *Fusarium* contamination had a strong adverse impact on the number of falls in wheat (*R* = −0.758). Similarly, Boyacioğlu and Hettiarachchy (1995) noted that *Fusarium* infection significantly reduced FN values in wheat grains. In line with our study, Papoušková et al. (2011) detected that FN values particularly decreased in contaminated samples. The results of this study showed that the observed quality changes in oats and triticale are closely related to changes in physicochemical and technological parameters caused by harvest delays and mycotoxin contamination, especially during cool and wet weather.

## 5 Conclusion

The results of our experiment showed that delaying harvesting in the presence of wet and cool weather conditions during the flowering and harvesting cereal periods leads to an increased risk of high levels of contamination of spring oats with mycotoxins DON, as well as triticale grain with DON and ZEA. Delayed harvesting also led to a reduction in the main quality indicators, including grain mass per hectoliter, FN, and viscosity. No effect of harvest time on T‐2 levels in oat and triticale grains was observed. The optimal harvest time, especially in wet weather conditions, is a crucial consideration for stable RVA pasting properties. The correlation analyses between mycotoxins and the quality and technological parameters of oats and triticale grains revealed a trend in which increasing mycotoxin contamination of spring cereal grains leads to negative consequences on mass per hectoliter, protein, starch, K, Ca, and Mg contents in oat grains and a reduction in mass per hectoliter, Mg, viscosity, and FN values in triticale grains. The relationship between mycotoxin contamination levels and the values of quality and technological parameters of late‐harvested oats and triticale indicates a potential negative economic impact on the food, feed, and bakery industries.

## Author Contributions

**Yuliia Kochiieru**: conceptualization, writing – original draft, methodology, data curation, formal analysis. **Audronė Mankevičienė**: conceptualization, supervision, methodology, writing – review and editing. **Akvilė Jonavičienė**: conceptualization, methodology. **Lauksmė Merkevičiūtė‐Venslovė**: conceptualization. **Eimantas Venslovas**: conceptualization, visualization, writing – review and editing. **Roma Semaškienė**: conceptualization, methodology. **Jūratė Ramanauskienė**: investigation, formal analysis. **Karolina Lavrukaitė**: investigation. **Mykola Kochiieru**: investigation, conceptualization, formal analysis. **Jurgita Cesevičienė**: conceptualization, methodology, supervision.

## Conflicts of Interest

The authors declare no conflicts of interest.
